# Impaired lysosomal acidity maintenance in acid lipase-deficient cells leads to defective autophagy

**DOI:** 10.1016/j.jbc.2024.105743

**Published:** 2024-02-12

**Authors:** Takahito Moriwaki, Seigo Terawaki, Takanobu Otomo

**Affiliations:** Department of Molecular and Genetic Medicine, Kawasaki Medical School, Kurashiki, Okayama, Japan

**Keywords:** autophagy, LIPA, lysosomal acidity, V-ATPase, ammonia

## Abstract

The lysosome is an acid organelle that contains a variety of hydrolytic enzymes and plays a significant role in intracellular degradation to maintain cellular homeostasis. Genetic variants in lysosome-related genes can lead to severe congenital diseases, such as lysosomal storage diseases. In the present study, we investigated the impact of depleting lysosomal acid lipase A (LIPA), a lysosomal esterase that metabolizes esterified cholesterol or triglyceride, on lysosomal function. Under nutrient-rich conditions, *LIPA* gene KO (*LIPA*^KO^) cells exhibited impaired autophagy, whereas, under starved conditions, they showed normal autophagy. The cause underlying the differential autophagic activity was increased sensitivity of *LIPA*^KO^ cells to ammonia, which was produced from l-glutamine in the medium. Further investigation revealed that ammonia did not affect upstream signals involved in autophagy induction, autophagosome–lysosome fusion, and hydrolytic enzyme activities in *LIPA*^KO^ cells. On the other hand, *LIPA*^KO^ cells showed defective lysosomal acidity upon ammonia loading. Microscopic analyses revealed that lysosomes of *LIPA*^KO^ cells enlarged, whereas the amount of lysosomal proton pump V-ATPase did not proportionally increase. Since the enlargement of lysosomes in *LIPA*^KO^ cells was not normalized under starved conditions, this is the primary change that occurred in the *LIPA*^KO^ cells, and autophagy was affected by impaired lysosomal function under the specific conditions. These findings expand our comprehension of the pathogenesis of Wolman's disease, which is caused by a defect in the *LIPA* gene, and suggest that conditions, such as hyperlipidemia, may easily disrupt lysosomal functions.

Lysosomes contain various acid hydrolases and are involved in degrading a wide range of cellular materials, such as proteins, lipids, nucleic acids, and intracellular organelles. Because lysosomes are essential for cell metabolism, their dysfunction is related to a variety of diseases known as lysosomal storage diseases (LSDs) (1, 2). LSDs are rare congenital metabolic disorders characterized by the accumulation of metabolites in lysosomes because of defects in lysosomal hydrolases, membrane proteins, lipid or ion transporters, and enzyme-modifying factors (3). Presently, more than 50 LSDs are known, and the pathology of each LSD varies depending on the responsible defective gene. Defects in lysosomal hydrolases are characterized by the continuous accumulation of specific target substrates. The accumulation of substrates in lysosomes directly or indirectly affects multifaceted lysosomal functions, such as vesicle trafficking, calcium homeostasis, inflammatory responses, and/or activation of cell death pathways.

Wolman’s disease or cholesterol ester storage disease is caused by a deficiency of lysosomal acid lipase A (LIPA) (4, 5). LIPA is a lysosomal hydrolase that converts cholesteryl ester into fatty acid and free cholesterol or breaks down triglycerides into free fatty acid and glycerol (6). Subsequently, cholesterol is transported from lysosomes to other organelles for recycling *via* NPC intracellular cholesterol transporter 1 (NPC1) (7). Dysfunction of the *NPC1* gene results in Niemann–Pick disease type C, characterized by widespread accumulation of free cholesterol throughout the body (8). Previous studies have demonstrated that autophagy is impaired in cells from Niemann–Pick disease type C patients caused by the disruption of fusion between autophagosomes and lysosomes (9, 10, 11). On the other hand, the effect of LIPA deficiency on autophagy has remained unclear. In the present study, we employed CRISPR–Cas9 to generate KO cells of the LIPA gene (*LIPA*^KO^ cells) to determine the effects of LIPA depletion on lysosome function and autophagic degradation.

## Results

### Impaired autophagy in *LIPA*^KO^ cells was restored under starved conditions

**Figure 1:**
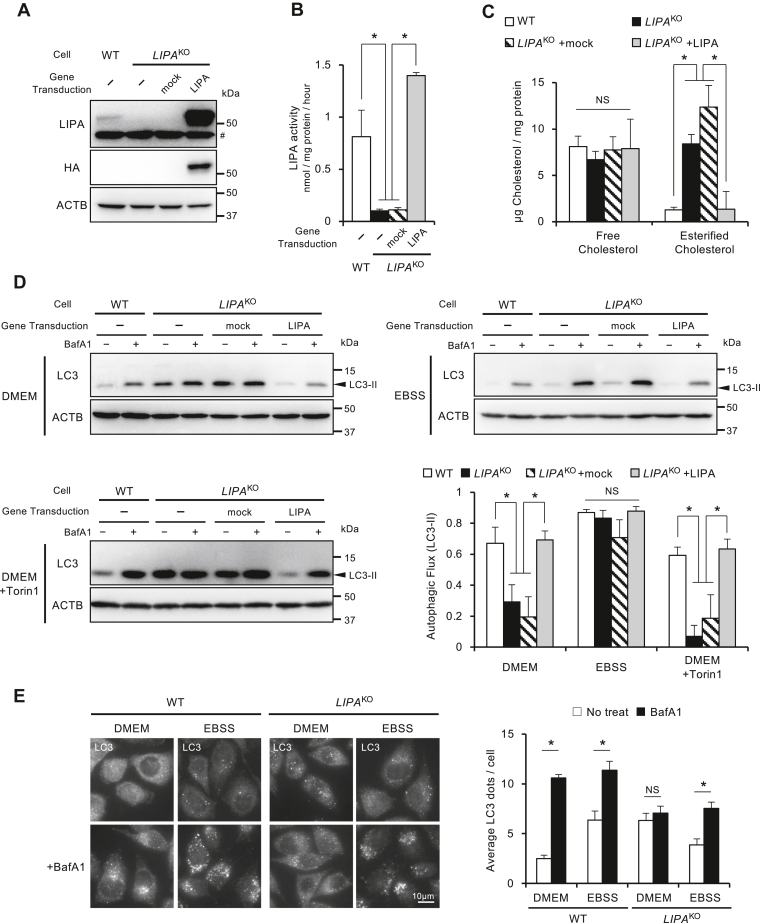
**Impaired autophagic flux under nutrient-rich conditions in *LIPA***^**KO**^**cells.***A*, determination of deficiency of LIPA protein in *LIPA*^KO^ cells and expression of LIPA-HA protein. LIPA and LIPA-HA proteins were determined by anti-HA or anti-LIPA antibodies by Western blotting. # indicates nonspecific bands. *B*, determination of reduced enzymatic activity in *LIPA*^KO^ cells. The enzyme activity of LIPA was assessed using 4MU palmitate. The difference in 4MU produced between the presence and absence of Lalist2 was considered as LIPA activity. *C*, measurement of accumulated cholesterol. Cellular cholesterol levels were measured. The amount of esterified cholesterol was calculated by the difference between cholesterol esterase–treated and nontreated samples. *D*, autophagic flux in *LIPA*^KO^ cells. Cells were cultured in DMEM, EBSS, or DMEM supplemented with 250 nM Torin1 in the absence or the presence of 125 nM BafA1 for 2 h. Then, LC3-II flux was assessed through Western blotting. Representative images and quantification results of LC3-II flux are presented. *E*, LC3-dot flux assay. Cells were stained with anti-LC3 and visualized by fluorescence microscopy. Typical images (*left*) and quantified results (*right*) were shown. Averaged LC3 dots count per cell were obtained from 10 pictures in three independent experiments. All graphs represent the mean and SD from three independent experiments. Statistical analyses were conducted using Student's *t* test or multiple comparison one-way ANOVA followed by post hoc Tukey's test (∗*p* < 0.05, NS *p* ≥ 0.05). 4MU, 4-methylumbelliferyl; BafA1, bafilomycin A1; DMEM, Dulbecco's modified Eagle's medium; EBSS, Earle's balanced salt solution; HA, hemagglutinin; LIPA, lysosomal acid lipase A; NS, not significant.

To investigate the effects of LIPA deficiency on autophagy and lysosomes, we generated KO cell lines of the *LIPA* gene in HeLa cells using the CRISPR–Cas9 system (Table. S1). Characterization of these KO cell lines was confirmed with depletion of LIPA protein (Fig. 1*A*), decreased LIPA enzyme activity (Fig. 1*B*), and accumulation of esterified cholesterol (Fig. 1*C*), which were rescued by hemagglutinin (HA)-tagged LIPA (LIPA-HA) expression. These observations indicate that the *LIPA*^KO^ cells have a similar phenotype to patients with Wolman’s disease.

Next, we investigated the autophagic function of these cells. LC3-II, a microtubule-associated protein 1 light chain 3 beta form II, localizes on the membrane of autophagic vesicles and is an established marker to assess autophagy (12). The difference in the amount of LC3-II with and without lysosome inhibitors reflects the amount of autophagic degradation at a given time of lysosome inhibitor administration. We treated cells with bafilomycin A1 (BafA1), an inhibitor of V-ATPase as a lysosomal inhibitor (13, 14, 15), and evaluated the autophagic degradation speed called autophagic flux.

As shown in Figure 1*D* (*upper left*), WT cells showed an increased amount of LC3-II with BafA1 treatment indicating normal autophagic flux. *LIPA*^KO^ cells showed similarly increased and no significant difference in the amount of LC3-II with and without BafA1 treatment. Interestingly, the autophagy defect of *LIPA*^KO^ cells was recovered upon starvation in Earle's balanced salt solution (EBSS) (Fig. 1*D*, *upper right*). These results indicate that autophagic flux is impaired in *LIPA*^KO^ cells under nutrient-rich conditions. Under nutrient-rich conditions, autophagy activity is suppressed to a minimum level by mechanistic target of rapamycin kinase (mTOR) signaling. Conversely, exposure to starved conditions triggers mTOR sensing of amino acid deprivation, resulting in the desuppression of autophagy machinery and an increase in autophagic flux (16). We observed that treatment with the mTOR inhibitor, Torin1, under nutritional conditions did not restore autophagy in *LIPA*^KO^ cells (Fig. 1*D*, *lower left*). From the results that *LIPA*^KO^ cells showed different reactions to starvation and mTOR inhibition, it is suggested that different mechanisms from mTOR signaling underlie the autophagic impairment in *LIPA*^KO^ cells.

We further examined basal autophagic activity using several different autophagy assays other than LC3-II flux. We tested the autophagic flux of adaptor proteins as alternative autophagy substrates: p62, tax1 binding protein 1, NCOA4 (nuclear receptor coactivator 4), and NDP52 (nuclear dot 10 protein 52, also known as CALCOCO2 [calcium binding and coiled-coil domain 2]) (Fig. S1*A*). Among these substrates, tax1 binding protein 1 and NCOA4 flux showed obvious autophagic inhibition in nutrient-rich conditions and recovery in starved conditions with *LIPA*^KO^ cells. The difference in autophagic flux of other substrates between WT and *LIPA*^KO^ cells was unclear. From these results, sensitivities of several autophagic substrates are variable. In the present study, we use HeLa cell lines and the anti-LC3 antibody from the MBL, where LC3-I detection is so weak with this combination. The antibody from Novus that could detect faint bands of LC3-I produced comparable results (Fig. S1*A*). Recently developed HaloTag-based autophagy flux assay is a highly sensitive, objective, and quantitative method (17). We established cells stably expressing HaloTag-conjugated LC3 (Halo-LC3) and examined basal autophagic change by the nutrient condition in *LIPA*^KO^ cells. Ligand-bound HaloTag (Halo^Ligand^) has been known to show tolerance to degradation in lysosomes. In *LIPA*^KO^ cells, Halo^Ligand^-LC3 hardly converted to Halo^Ligand^ under Dulbecco's modified Eagle's medium (DMEM) but converted comparably to WT cells under EBSS (Fig. S1*B*). We also employed another technique to assess autophagy. The tfLC3 (tandem fluorescence-tagged LC3) construct that comprises LC3 tandemly fused with red fluorescent protein (RFP) and green fluorescent protein (GFP) is used to verify autophagosome–lysosome fusion (18, 19). Since GFP is rather quenched in the acidic environment than RFP, acidic autolysosomes are marked more strongly by RFP than by GFP (appears as *red puncta*), whereas autophagosomes are marked by both GFP and RFP (appears as *yellow puncta*). Under the nutrient-rich condition, the number of RFP^+^GFP^+^ puncta significantly increased in *LIPA*^KO^ cells than in WT cells, which indicates impairment of autophagy in *LIPA*^KO^ cells. On the other hand, under the starved condition, the numbers of puncta were comparable between WT and *LIPA*^KO^ cells (Fig. S1*C*).

The recovery of autophagy in *LIPA*^KO^ cells under the starved condition was confirmed in different clones of *LIPA*^KO^ (Fig. S1, *D* and *E*) and by LC3-dot flux (Fig. 1*E*). Altogether, it is confirmed that *LIPA*^KO^ cells show impaired autophagic activity under nutrient-rich condition, which was restored to the normal level by starvation.

### *LIPA*^KO^ cells had increased sensitivity to ammonia resulting in inhibition of autophagy

**Figure 2:**
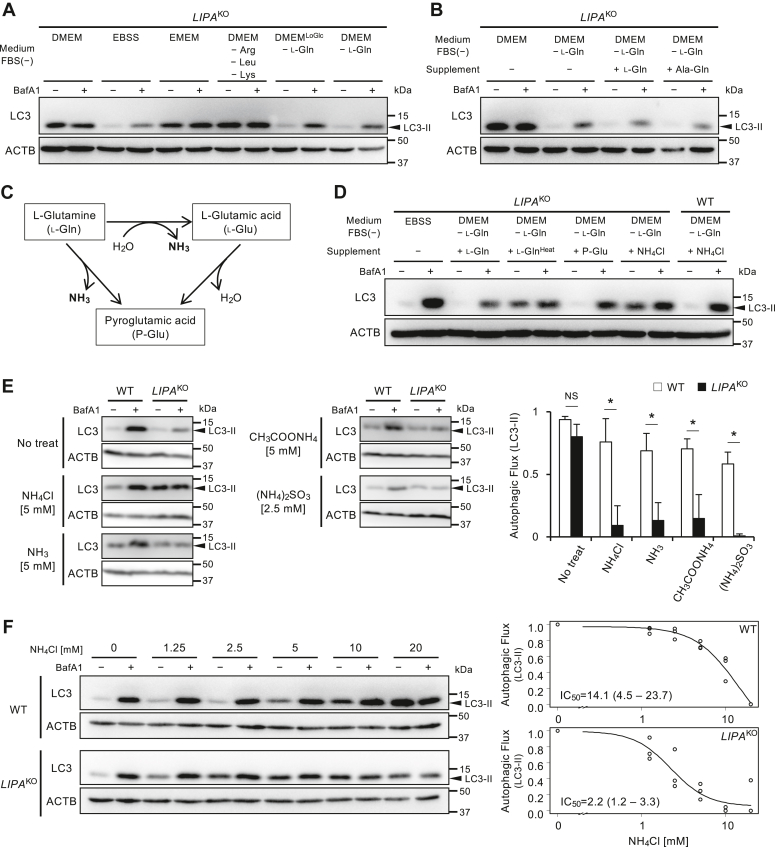
**Autophagic inhibition by ammonia produced *via*****l****-glutamine (****l****-Gln) degradation in DMEM.***A* and *B*, determination of l-Gln effects on autophagic inhibition in *LIPA*^KO^ cells. Cells were cultured under various nutrient conditions, as indicated, for 2 h, and LC3-II flux was analyzed through Western blotting. In addition, 4 mM l-Gln or l-Alanyl-l-glutamine (Ala-Gln) was included in the cell culture, as indicated in *B*. *C*, schematic representation of l-Gln pyrolysis process. l-Gln undergoes conversion to ammonia and pyroglutamic acid (P-Glu) through pyrolysis. *D*, determination of degradation products of l-Gln for autophagic inhibition in *LIPA*^KO^ cells. Cells were cultured in l-Gln-depleted DMEM, supplemented with or without 4 mM l-Gln or its derivatives for 2 h. Heat-treated l-Gln (95 °C, 4 h) is denoted as “l-Gln^Heat^.” The effects of these supplements on autophagy were assessed using the LC3-II flux assay. *E*, similar effect of other ammonium compounds. Cells were treated with or without 5 mM NH_4_Cl, 5 mM NH_3_, 5 mM CH_3_COOH, or 2.5 mM (NH_4_)_2_SO_3_ in EBSS for 2 h. Subsequently, LC3-II flux was assessed through Western blotting. Representative images (*left*) and quantification results (*right*) are presented. The graph represents the mean and SD from three independent experiments. Statistical analyses were conducted using multiple comparison one-way ANOVA followed by post hoc Tukey’s test (∗*p* < 0.05, NS *p* ≥ 0.05). *F*, determination of IC_50_ for NH_4_Cl. Cells were treated with NH_4_Cl at the indicated doses in EBSS for 2 h, and LC3-II flux was assessed through Western blotting. Representative images (*left*) and a four-parameter log-logistic curve from three independent experiments (*right*) are provided. *Open circles* represent relative LC3-II flux at each concentration of NH_4_Cl in three independent experiments. DMEM, Dulbecco's modified Eagle's medium; EBSS, Earle's balanced salt solution; LIPA, lysosomal acid lipase A; NH_4_Cl, ammonium chloride; NS, not significant.

To elucidate the mechanism underlying autophagic impairment in *LIPA*^KO^ cells, we aimed to identify the key factors involved in the contents of culture mediums. We tested with *LIPA*^KO^ cells in different culture conditions and investigated autophagic flux. LC3 flux did not recover with either Eagle’s minimal essential medium or DMEM deprived of arginine (Arg), leucine (Leu), and lysine (Lys). Conversely, autophagy was reinstated when *LIPA*^KO^ cells were exposed to DMEM lacking l-Gln, irrespective of glucose levels (Fig. 2*A*). This suggests that l-Gln triggers autophagic arrest in *LIPA*^KO^ cells. We supplemented the l-Gln-depleted medium with l-Gln or l-alanyl-l-glutamine (Ala-Gln), a stable l-Gln source (20), to assess potential autophagy suppression. Unexpectedly, the addition of l-Gln or Ala-Gln to l-Gln-depleted DMEM did not repress autophagy in *LIPA*^KO^ cells (Fig. 2*B*).

To address these contradictory findings, we explored the hypothesis that degradation products of l-Gln impact autophagic activity. l-Gln is known to either directly convert to pyroglutamic acid through heat or undergo hydrolysis to l-glutamic acid, and during the process of glutamine metabolism, ammonia is also produced (Fig. 2*C*) (21, 22). Autophagic impairment in *LIPA*^KO^ cells was observed upon the addition of l-Gln heated at 95 °C for 4 h (l-Gln^Heat^) to l-Gln-depleted DMEM.

This suggests that the pyrolysis product of l-Gln contributes to autophagy inhibition. Pyroglutamic acid supplementation on l-Gln-depleted medium did not impair autophagic flux, and ammonium chloride (NH_4_Cl) halted autophagic flux (Fig. 2*D*). Importantly, the suppressive effect of NH_4_Cl on autophagy in *LIPA*^KO^ cells was not replicated in WT HeLa-Kyoto cells (Fig. 2*D*).

To reveal whether the inhibitory impact on autophagy in *LIPA*^KO^ cells is exclusive to NH_4_Cl, other ammonia derivatives, including ammonia (NH_3_), ammonium acetate (CH_3_COONH_4_), and ammonium sulfate ((NH4)_2_SO_3_), were tested. At equivalent ammonium ion concentrations, autophagic flux was similarly impaired in *LIPA*^KO^ cells by ammonia derivatives (Fig. 2*E*). From these results, it is suggested that ammonia plays a key role in autophagy in *LIPA*^KO^ cells.

We performed an LC3 flux assay on WT and *LIPA*^KO^ cells treated with varying NH_4_Cl concentrations to evaluate their ammonia sensitivity. The relative autophagy activity against each ammonia concentration was plotted, and 50% inhibition concentration (IC_50_) was calculated using a four-parameter log-logistic curve. While high NH_4_Cl concentrations impaired autophagic flux in WT cells, a significant difference in IC_50_ was evident between WT (IC_50_ = 14.1 [95% confidence interval (95% CI): 4.5–23.7] [mM]) and *LIPA*^KO^ cells (IC_50_ = 2.2 [95% CI: 1.2–3.3] [mM]) (Fig. 2*F*).

### Autophagic induction and autophagosome–lysosome fusion are not influenced by ammonia in *LIPA*^KO^ cells

We then proceeded to investigate the mechanism by which ammonia inhibits autophagy in *LIPA*^KO^ cells. Autophagy progression is governed by multistep biological cascades: (i) nucleation and extension of isolation membrane upon autophagic induction signaling to create autophagosome, (ii) fusion between autophagosomes and lysosomes, resulting in autolysosome formation, and (iii) degradation of autophagic substrates within autolysosomes (23).

We first focused on the upstream signaling that induces autophagy. The initiation of autophagosome formation is highly dependent on unc-51 like autophagy activated kinase 1 (ULK1), which is competitively regulated by the protein kinase AMP-activated catalytic subunit alpha 1 (AMPK) and mTOR, which reflect intracellular nutrient status (Fig. S2*A*). We examined the phosphorylation level of mTOR and AMPK to assess the activation status of these kinases under NH_4_Cl treatment. AMPK phosphorylation was significantly enhanced after 5 mM NH_4_Cl treatment (Fig. S2*B*), and no significant change in mTOR phosphorylation was observed after NH_4_Cl (Fig. S2*C*). This is consistent with previous reports (24). It has also been reported that BafA1 can be an inhibitor of mTOR, but its effect would be negligible (25). Further examination of ULK1 phosphorylation revealed that phosphorylation of Ser^555^ of ULK1, a target of AMPK and contributor to autophagy activation, was enhanced by ammonia, but phosphorylation of Ser^757^ of ULK1, a target of mTOR and suppressor of autophagy, was not affected (Fig. S2*D*). This result is consistent with the activation status of AMPK and mTOR, with no significant difference between WT and *LIPA*^*KO*^ cells (Fig. S2, *B* and *C*). This indicates that treatment with NH_4_Cl did not impair the autophagy initiation signal. Therefore, we conclude that the autophagy inhibitory effect of NH_4_Cl in *LIPA*^KO^ cells is not associated with upstream signaling.

**Figure 3:**
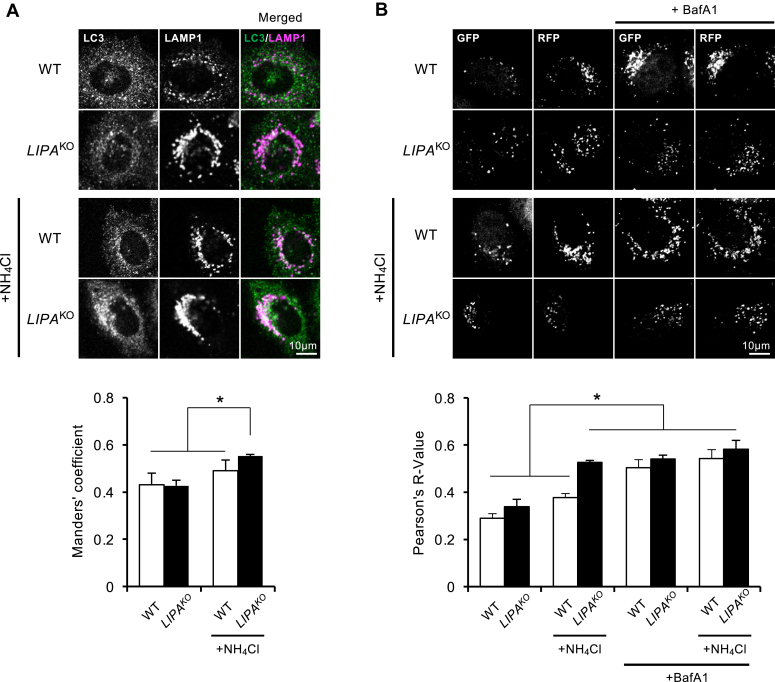
**Increased sensitivity of lysosomes to ammonia in *LIPA***^**KO**^**cells.***A*, determination of autophagosome–lysosome fusion by immunocytochemistry. WT and *LIPA*^KO^ cells were cultured with or without 5 mM NH_4_Cl and protease inhibitors (10 μg/ml E64d and 10 μg/ml pepstatin A) in EBSS for 2 h. Endogenous LC3 and LAMP1 were analyzed using immunocytochemistry, and representative cell images are displayed. Colocalization between LC3 and LAMP1 was assessed using Manders' coefficient (LC3 over LAMP1). *B*, determination of autophagosome–lysosome fusion by tfLC3. Cells stably expressing tfLC3 were treated with or without 5 mM NH_4_Cl and/or 125 nM BafA1 in EBSS for 2 h. Representative images of GFP and RFP signals for each condition are presented. Colocalization of GFP and RFP signals was evaluated using the Pearson's correlation coefficient (*R* value) between the two signals. All graphs represent the mean and SD from three independent experiments. Statistical analyses were conducted using multiple comparison one-way ANOVA followed by post hoc Tukey's test (∗*p* < 0.05). BafA1, bafilomycin A1; EBSS, Earle's balanced salt solution; GFP, green fluorescent protein; LAMP1, lysosomal-associated membrane protein 1; LIPA, lysosomal acid lipase A; NH_4_Cl, ammonium chloride; RFP, red fluorescent protein; tfLC3, tandem fluorescence-tagged LC3.

Next, we investigate autophagosome–lysosome fusion. To assess the fusion process, we cultivated both WT and *LIPA*^KO^ cells in the presence of protease inhibitors to prevent lysosomal degradation of LC3 while preserving lysosomal pH. Subsequently, these cells were treated with or without 5 mM NH_4_Cl in EBSS, followed by immunostaining with LC3 and lysosomal-associated membrane protein 1 (LAMP1) antibodies to visualize autophagosomes and lysosomes, respectively. NH_4_Cl treatment did not reduce the colocalization between LC3 and LAMP1 in all experimental conditions (Fig. 3*A*), suggesting that the impairment of autophagy in *LIPA*^KO^ cells did not originate from defects in autophagosome–lysosome fusion. The substantial increase in colocalized LC3 and LAMP1 signals in *LIPA*^KO^ cells can be attributed to an elevated number of undegraded autolysosomes.

To assess autophagosome–lysosome fusion, we performed the tfLC3 assay. WT cells, the GFP signal was much weaker than the RFP signal, both in the absence and presence of 5 mM NH_4_Cl in EBSS, indicating effective autophagosome–lysosome fusion (Fig. 3*B*). As a negative control, treatment of WT cells with BafA1 restored the quenched GFP signal and increased the Pearson’s correlation coefficient (*R* value) between GFP and RFP signals (Fig. 3*B*), confirming the proper localization of the tfLC3 probe to the acidic compartment after autophagosome–lysosome fusion. NH_4_Cl treatment increased the *R* value to levels comparable to its BafA1-treated controls in *LIPA*^KO^ cells (Fig. 3*B*), which indicates GFP was not quenched.

The interpretation for the results of the tfLC3 assay is that autophagosome–lysosome fusion is impaired and/or autolysosomal acidity is disrupted after fusion in ammonia-treated *LIPA*^KO^ cells. Combined with the results that *LIPA*^KO^ cells showed comparable colocalization between LC3 and LAMP1 to WT cells (Fig. 3*A*), ammonia treatment probably disturbs the acidic environment of lysosomes–autolysosomes specifically in *LIPA*^KO^ cells.

### Lysosomal pH is increased in *LIPA*^KO^ cells

**Figure 4:**
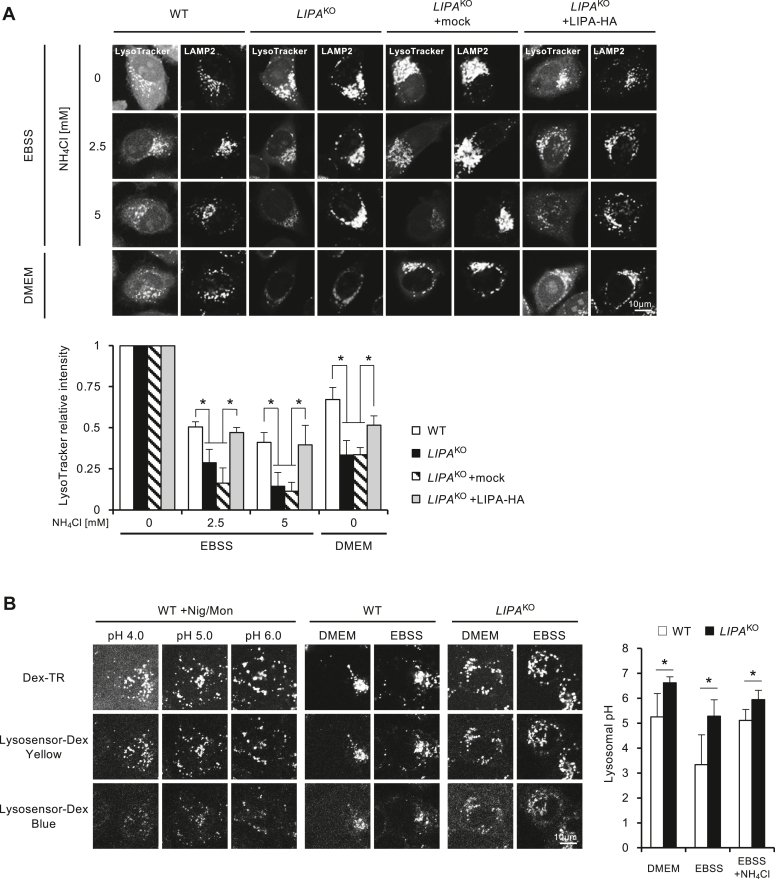
**Enlarged lysosomes but unchanged levels of V-ATPase in *LIPA***^**KO**^**cells.***A*, determination of decreased lysosomal pH because of NH_4_Cl treatment or culture in DMEM. Cells were treated with 0, 2.5, or 5 mM NH_4_Cl in EBSS or cultured in DMEM for 2 h. Lysosomes were stained with LysoTracker *Red* for 30 min, followed by LAMP2 immunostaining. The relative LysoTracker intensities on lysosomes (LAMP2 positive) were quantified using ImageJ. Image analysis was conducted on a minimum of 10 pictures in each condition. *B*, pH measurement by LysoSensor *Yellow*/*Blue* dextran, 10,000 MW. Cells were seeded on glass bottom dishes and treated with 0.5 mg/ml LysoSensor *Yellow*/*Blue* dextran, 10,000 MW, and 0.1 mg/ml Dextran, Texas Red, 3000 MW for 24 h. Then, cells were cultured in DMEM, EBSS, or EBSS containing 5 mM NH_4_Cl for 2 h. The ratio of intensities of *yellow* and *blue* signals was quantified using ImageJ. Image analysis was conducted on a minimum of 10 pictures in each condition. All graphs represent the mean and SD from three independent experiments. Statistical analyses were conducted using multiple comparison one-way ANOVA followed by post hoc Tukey's test (∗*p* < 0.05). DMEM, Dulbecco's modified Eagle's medium; EBSS, Earle's balanced salt solution; LAMP2, lysosomal-associated membrane protein 2; LIPA, lysosomal acid lipase A; MW, molecular weight; NH_4_Cl, ammonium chloride.

We examined the lysosomal acidity in different conditions in *LIPA*^KO^ cells. We stained cells with LysoTracker, a fluorescent dye that accumulates in acidic compartments in a pH-dependent manner, together with immunostaining of lysosomal-associated membrane protein 2 (LAMP2). The amount of lysosome differs between WT and *LIPA*^KO^ cells and may change by culture conditions. Accumulation of LysoTracker was normalized by the amount of LAMP2. NH_4_Cl treatment progressively attenuated the LysoTracker signal on LAMP2-positive vesicles in a dose-dependent manner, indicating elevated lysosomal pH. This shift was observed across all cell types, with the effect being more pronounced by the KO of *LIPA* genes and recovered by expression of LIPA-HA (Fig. 4*A*). Lysosomal pH was also assessed with a different pH indicator, LysoSensor. Using this probe, lysosomal pH values in WT and *LIPA*^KO^ cells were measured as 5.24 and 6.62 (under DMEM), 3.33 and 5.28 (under EBSS), 5.11 and 5.94 (under NH_4_Cl-containing EBSS), respectively. These results indicate that *LIPA*^KO^ cells show higher pH values than WT cells. In addition, *LIPA*^KO^ cells show low pH under EBSS (*i.e.*, the absence of ammonia) that is comparable to WT cells in DMEM, which may confer normal autophagic function in starved *LIPA*^KO^ cells (Fig. 4*B*).

V-ATPase is known to play a central role in proton uptake and subsequent lysosomal acidification (26, 27). To gain deeper insights into the molecular mechanisms underlying how LIPA deficiency interferes with pH maintenance mechanisms and alters the sensitivity to pH-disturbing agents, we assessed the response to BafA1, a V-ATPase inhibitor. WT and *LIPA*^KO^ cells were exposed to various concentrations of BafA1, and the IC_50_ for BafA1 was determined based on autophagic flux. Unexpectedly, no discernible differences (with overlapping 95% CIs) were observed in IC_50_ values between WT (IC_50_ = 9.2 [95% CI: 1.6–16.8] [nM]) and *LIPA*^KO^ cells (IC_50_ = 7.3 [95% CI: 3.8–10.9] [nM]) (Fig. S3*A*). Inhibiting V-ATPase by various concentrations of BafA1 similarly inhibits LysoTracker accumulation in WT and *LIPA*^KO^ cells (Fig. S3*B*). These results suggest that the function of V-ATPase, which may be affected by the subunit localization on lysosome, formation of the pump complexes, or Vo–V1 assembly, is comparable between WT and *LIPA*^KO^ cells.

Lysosomal degradation of autophagic substrates is dependent not only on the lysosomal acidic environment but also on lysosomal hydrolases (lysosomal enzymes). Lysosomal enzyme activities were retained in *LIPA*^KO^ cells at least ∼50% of the WT for all measured enzymes (Fig. S3). Since LSDs are recessive diseases, we believe these activities of lysosomal enzymes are not substantially diminished and are considered sufficient for maintaining lysosomal function.

To summarize, *LIPA*^KO^ cells exhibited normal autophagic induction, autophagosome–lysosome fusion, and lysosomal enzyme levels. The function of V-ATPase was not changed in *LIPA*^KO^ cells; however, autophagic flux seemed to be impaired by increased lysosomal pH under ammonia loading. These results suggest that the capacity for maintaining lysosomal acidity is reduced in *LIPA*^KO^ cells.

### Lysosomes are enlarged in *LIPA*^KO^ cells

**Figure 5:**
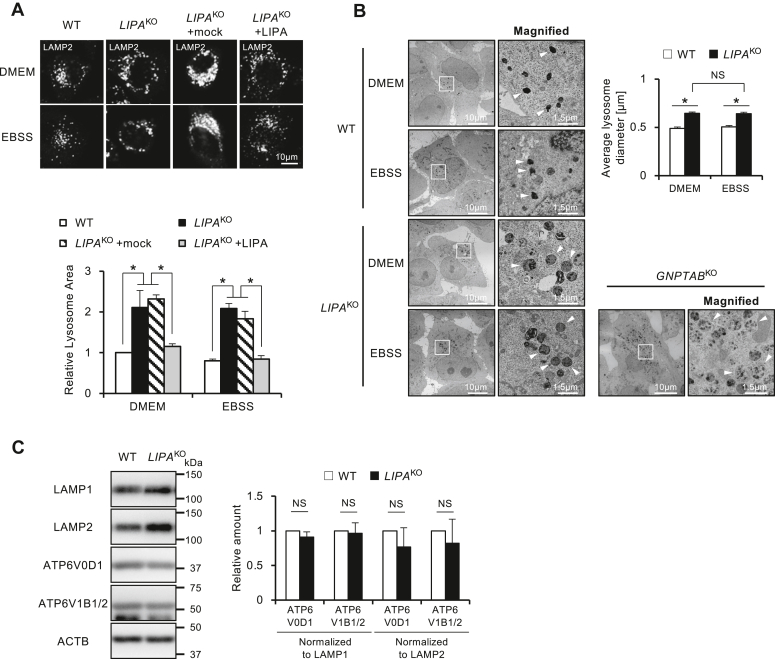
**Enlarged lysosomes but unchanged levels of V-ATPase in *LIPA***^**KO**^**cells.***A*, measurements of average lysosome area. Cells were cultured in DMEM or EBSS for 2 h. Lysosomes were stained with LAMP2 immunostaining and measured average lysosomal area. Lysosomal size was normalized to the size of WT under DMEM. A total of 10 pictures were analyzed in each condition. The graph represents the mean and SD from three independent experiments. *B*, measurements of average lysosome diameter by electron microscopy. Cells were observed by electron microscopy, and lysosome diameter was measured. Lysosomes are indicated by *arrowheads*. At least 50 lysosomes were analyzed in each condition. The graph represents the mean and SD of measured lysosomes. *C*, evaluation of V-ATPase. Lysosomal membrane proteins were analyzed by Western blotting. The ratios of ATP6V0D1 or ATP6V1B1/2 to LAMP1 or LAMP2 were calculated. The graphs represent the mean and SD from three independent experiments. Statistical analyses were conducted using multiple comparison one-way ANOVA followed by post hoc Tukey's test (∗*p* < 0.05, NS *p* ≥ 0.05). DMEM, Dulbecco's modified Eagle's medium; EBSS, Earle's balanced salt solution; LAMP1, lysosomal-associated membrane protein 1; LAMP2, lysosomal-associated membrane protein 2; LIPA, lysosomal acid lipase A; NS, not significant.

Microscopic observation revealed that LAMP2 staining showed a tendency for increased lysosomal size in *LIPA*^KO^ cells compared with WT. Therefore, we performed a quantitative assessment of the lysosomal size. We immunostained cells with LAMP2 and measured the average area of individual lysosome vesicles. Results indicated a significant increase in lysosome size in *LIPA*^KO^ cells under both nutrient-rich and starved conditions compared with the WT (Fig. 5*A*). Enlargement of lysosomal size was restored by LIPA-HA rescue in *LIPA*^KO^ cells (Fig. 5*A*). For a more detailed analysis, we observed lysosomes under electron microscopy. The diameter of lysosomes was significantly larger in *LIPA*^KO^ cells than that of WT (Fig. 5*B*), which is consistent with the results of immunostaining.

Lysosomal sizes did not differ between nutrient-rich and starved conditions in *LIPA*^KO^ cells. *LIPA*^KO^ cells show autophagic impairment in nutrient-rich (*i.e.*, ammonia-loaded) conditions but normal autophagic flux in starved (*i.e.*, ammonia-free) conditions. Taken together, the enlargement of lysosomes observed in *LIPA*^KO^ cells is a primary change in this cell line, not caused by the accumulation of storage materials induced by autophagy inhibition. The observation that the electron density of lysosomes was lower in *LIPA*^KO^ cells than WT (Fig. 5*B*) may also be a support.

The increased lysosome volume necessitates more proton pumps to uphold lysosomal acidity. In larger lysosomes in *LIPA*^KO^ cells, more proton pumps would be required to maintain the same acidic capacity as WT. V-ATPase comprises a peripheral V1 domain with eight subunits and an internal V0 domain with six subunits (28). In the present study, we analyzed amounts of ATPase H^+^ transporting V0 subunit d1 (ATP6V0D1) and ATPase H^+^ transporting V1 subunit B1 (ATP6V1B1), commonly used indicators of V-ATPase abundance. Representative lysosomal membrane markers, including LAMP1 and LAMP2, were elevated in *LIPA*^KO^ cells. In contrast, ATP6V0D1 and ATP6V1B per lysosome were comparable to those of the WT, as standardized by LAMP1 and LAMP2 (Fig. 5*C*).

Though both the function of V-ATPase (Fig. S3) and abundance of V-ATPase per lysosomal membrane area (Fig. 5*C*) are similar between WT and *LIPA*^KO^ cells, an increase in lysosomal diameter leads to an increase in volume to the third power in *LIPA*^KO^ cells. The compromised capacity of lysosomes to maintain acidity in *LIPA*^KO^ cells may primarily be attributed to the increased lysosomal size in *LIPA*^KO^ cells, which requires a greater amount of V-ATPase to sustain normal lysosomal acidification compared with WT cells.

### Cholesterol treatment mimics the lysosomal phenotype of the deficiency of LIPA

To consider the possibility that the lysosomal phenotype observed in the *LIPA*^KO^ cells was caused by impaired cholesterol metabolism, we observed cholesterol-treated WT cells. Cholesterol-treated cells showed impaired autophagic flux under DMEM or Torin1-containing DMEM but normal autophagic flux under EBSS (Fig. S5, *A* and *B*). In addition, autophagy flux was inhibited by 5 mM ammonia in cholesterol-treated cells (Fig. S5*C*), and these cells showed significantly higher ammonia sensitivity (IC_50_ = 3.2 [95% CI: 2.3–4.1] [mM]) compared with the cholesterol-untreated WT cells (no overlapping of 95% CI) (Fig. S5*D*).

We found that the LysoTracker signal in cholesterol-treated cells was more greatly reduced by ammonia than in the WT strain (Fig. S6*A*), suggesting that sensitivity to ammonia in cholesterol-treated cells was due to the decreased maintenance capacity of lysosomal acidity. In addition, 24-h cholesterol treatment increased the average lysosomal area 1.5 times, similar to *LIPA*^KO^ cells (Fig. S6*B*). These results indicate that cholesterol treatment mimics the pathophysiology of *LIPA*^KO^ cells.

## Discussion

The degradation of various kinds of substrates by lysosomes considerably contributes to the maintenance of cell homeostasis. It is well known that defective lysosomal degradation directly results in diseases like LSD (1, 2). In this study, we investigated the effects of acid lipase deficiency, which is involved in lipid metabolism in lysosomes, on autophagy function using HeLa cells. As a result, it was found that autophagy degradation was significantly impaired in *LIPA*^KO^ cells, but it was restored under starvation conditions. Finally, we have revealed that this recovery of autophagic degradation was caused by the removal of ammonia included in DMEM and not related to the well-known mechanisms of starvation-induced autophagy activation (16).

It has been reported that ammonia activates AMPK *via* the dopamine receptor D3 (29). AMPK activation was indeed observed in our experiments; however, there was no significant difference in AMPK phosphorylation levels between WT and *LIPA*^KO^ cells. Furthermore, there was also no difference in phosphorylation levels of ULK1 Ser^555^, which is the target of AMPK and is involved in autophagy activation (Fig. S2). This suggests that ammonia does not affect autophagy activity through modulating intracellular signaling. On the other hand, we noticed that the LysoTracker signal was apparently weaker in *LIPA*^KO^ cells than in WT cells (Fig. 4*A*). Since LysoTracker emits signals by accumulating in acidic compartments (30), we speculated that the reduction of its signal might indicate a decrease in lysosome acidity. In fact, *LIPA*^KO^ cells represent higher pH than WT cells under all conditions tested, when accurate lysosomal pH was determined using LysoSensor. In addition, *LIPA*^KO^ cells showed close to neutral pH (∼pH 6.0) in DMEM or EBSS supplemented with NH_4_Cl, whereas WT cells kept weak acidic conditions (∼pH 5.0) (Fig. 4*B*). Lysosome hydrolases function at low pH and show activity when transported into lysosomes (31). When combined with the LC3 flux data (Figs. 1 and 2), it indicates that the lysosomal degradation mechanism is still functioning at around pH 5.0 while the flux stops above pH 6.0. This suggests that there is a threshold for lysosomal enzyme activity between pH 5.0 and pH 6.0 (around pH 5.5). *In vitro* enzyme activities of *LIPA*^KO^ cells were not affected, which means the enzyme transportation to lysosome was normal (Fig. S4). Collectively, we presumed that ammonia increased the pH of lysosomes in *LIPA*^KO^ cells, resulting in a decrease in the activity of hydrolases and impaired the degradation function of lysosomes. Since the impairment of autophagic activity in *LIPA*^KO^ cells was observed by either ammonia or multiple ammonia salts (Fig. 2, *D* and *E*), it is definite that ammonium ion (NH_4_^+^) impairs the lysosomal acid maintenance mechanism of *LIPA*^KO^ cells. It is not clear whether it is an NH_4_^+^-specific effect or whether other neutralizing factors such as basic substances can give similar effects. Given that ammonia is produced as a result of amino acid catabolism, ammonia is probably the most common basic substance that actually affects lysosomal function *in vivo*.

By the microscopic analyses, we found that *LIPA*^*KO*^ cells were occupied with aberrant enlarged lysosomes than those of WT cells (Fig. 5, *A* and *B*). Increased lysosome size has been reported in connection with lysosomal diseases (32, 33, 34). In mucolipidosis II (also called I-cell disease), which is caused by biallelic pathogenic variants of the *GNPTAB* gene, transport of major lysosomal hydrolases to lysosomes is impaired (35). It is known that *GNPTAB*-deficient cells show an accumulation of undegraded substrates inside lysosomes and increased size of lysosomes by the defect of lysosomal degradation (Fig. 5*B*) (36). However, in *LIPA*^KO^ cells, no change in lysosome size was observed even after restoring autophagy function under EBSS conditions and promoting substrate degradation (Fig. 5, *A* and *B*). Therefore, it suggests that the increase in lysosomal size in *LIPA*^KO^ cells is not because of substrate accumulation. When comparing the two with electron microscope images, *LIPA*^KO^ cells observe large lysosomes with low electron density, whereas *GNPTAB*^*KO*^ cells show multiple electron-dense vesicles incorporated inside lysosomes; these two were obviously different (Fig. 5*B*, *lower right*).

The enlargement of lysosomes was reproduced by loading cholesterol from outside the cells (Fig. S6*B*). Thus, the alteration of cholesterol metabolites might be the cause of the increase in lysosomal size. *NPC1* and *NPC2*, the causative genes of Niemann–Pick disease, are responsible for the transport of free cholesterol in lysosomes (37). It has been reported that autophagosome–lysosome fusion is inhibited in this disease (9, 10, 11). It is common with *LIPA*^KO^ cells in terms of abnormalities in lipid metabolism; however, *LIPA*^KO^ cells have normal autophagosome–lysosome fusion (Fig. 3) in the present study. From this point of view, *LIPA-* and *NPC1/2*-deficient cells are considered to have different properties from each other. How changes in lipid composition are involved in the regulation of lysosomal size needs to be elucidated in future studies.

From our observations, the diameter of lysosomes in *LIPA*^KO^ cells increased to about 1.5 times that of WT cells (Fig. 5*B*). By calculation, this suggests that lysosomes of *LIPA*^KO^ cells have approximately twice the surface area, which is consistent with Figure 5*A*, and ∼3.7 times volume compared with those of WT cells. On the other hand, lysosomes of *LIPA*^KO^ cells had a comparable amount of V-ATPase per lysosomal membrane area to WT cells (Fig. 5*C*). Even if the V-ATPase per unit area of the lysosomal surface is equivalent, it is necessary to incorporate about 1.8 times more protons (H^+^) per V-ATPase considering the volume ratio. If there is no substrate degradation load, *LIPA*^KO^ cells may have a pH approaching the same level as WT cells over time, but in fact, the degradation substrate is also thought to consume H^+^ (38). The addition of ammonia may have exceeded the threshold for the allowable amount of acidification maintenance mechanism in *LIPA*^KO^ cells. In fact, not only V-ATPase but also opposing ion transporters such as CIC-7 and NHE, K^+^ channels TMEM175, and SLC family molecules that are transporters of organic substances, such as sugars and amino acids, work in cooperation with the acidification maintenance mechanism of lysosomes, and these are thought to contribute to the regulation of lysosomal size as well as pH (39). However, V-ATPase is the major factor for H^+^ uptake. The decrease in acidic ability in *LIPA*^KO^ cells is mainly because of an increase in lysosomal capacity in *LIPA*^KO^ cells, and the relative decrease in H^+^ pump expression level per lysosome volume is considered to be a rate-limiting condition, impairing resistance to ammonia.

Wolman’s disease, caused by biallelic pathogenic variants in the *LIPA* gene, exhibits a variety of symptoms, including an enlarged liver and failure, splenomegaly, anemia, low muscle tone, and developmental delay. This study revealed that ammonia reduces lysosomal function in *LIPA*^KO^ cells. As mentioned previously, ammonia is produced in the body by amino acid metabolism in peripheral tissues, transported to the liver, and converted to harmless urea by the ornithine cycle (40, 41). From the observation in rats, normal blood ammonia concentration was 2.9 ± 0.1 μg/ml, whereas 39.2 ± 1.8 μg/g tissue in the liver, indicating ∼13 times higher than blood (42). Elevated ammonia levels in local tissue, such as the liver, may disturb normal autophagic function and lead to organ dysfunction in Wolman's disease.

In our study, even when WT cells were cultured with about twice as much excess cholesterol as normal, the same phenotype as *LIPA*^KO^ cells was reproduced, such as a decrease in LC3 flux, increased sensitivity to ammonia, and enlarged lysosomes (Figs. S5 and S6). These results indicate that abnormalities in cholesterol metabolism can lead to loss of lysosomal degradative function. Indeed, it has been reported that even in the absence of lysosomal gene abnormalities in humans, acquired lysosomal dysfunction can occur because of excessive dietary lipid intake, certain medications, and frequent plasmapheresis (43, 44, 45, 46). Abnormal lipid metabolism and nitrogen overload may contribute to lysosomal dysfunction.

In addition, our results have important implications for autophagy research. DMEMs are widely used in cell culture, and usual DMEMs contain 4 mM l-Gln. Because l-Gln is known to be unstable (47, 48), l-Gln supplements are often added to DMEMs just before use. In this study, it was shown that ammonia produced by the thermal decomposition of l-Gln affects autophagy activity under specific conditions. This means that when conducting lysosome-related studies, attention should be paid to media warming and l-Gln supplementation.

In summary, we have clarified that the pH maintenance mechanism of lysosomes is disrupted when lysosomal lipid-metabolizing enzymes are deficient in the present study. Lysosomes are involved in various cellular functions, such as endocytosis, signal transduction regulation, metabolic regulation, and membrane transport in addition to autophagy (49, 50). Disruption of pH homeostasis in lysosomes can affect conditions, such as cancer, neurodegenerative diseases, and aging, and pose higher health risks than previously recognized. To understand the mechanisms of these diseases and establish therapeutic strategies, further elucidation of the mechanism of disruption of lysosomal pH homeostasis caused by abnormal cholesterol metabolism is required.

## Experimental procedures

### Cell culture

HeLa-Kyoto cells were cultured in DMEM (Sigma–Aldrich; catalog no.: D5796) supplemented with 10% fetal bovine serum and 1× penicillin–streptomycin (Nacalai Tesque; catalog no.: 26252-94) in a 5% CO_2_ atmosphere at 37 °C. Cells were treated with chemicals at the concentration below unless specifically noted: 125 nM BafA1, 10 μg/ml E64d, 10 μg/ml pepstatin A, 10 nM nigericin, and 10 nM monensin.

### Generation of KO cells

To establish KO cells, guide sequences were designed using the Benchling CRISPR Guide RNA Design Tool (https://www.benchling.com/crispr/). The guide sequences were then cloned into the pSpCas9 (BB)-2A-GFP (px458) vector. px458 was obtained as a gift from Feng Zhang (Addgene; catalog no.: 48138). The px458 plasmid was transfected into cells using Effectene Transfection Reagent (Qiagen; catalog no.: 301425). After 48 h of transfection, GFP-positive populations were sorted into single-cell cultures using a BD FACS Aria III Cell Sorter (BD Biosciences) and cultured in 96-well plates for 2 weeks. Genomic DNA from the clones was purified using QuickExtract DNA extraction solution (Lucigen; catalog no.: QE0905T) and analyzed by Sanger sequencing (Table S1).

### Stable gene expression with viral transduction

LIPA complementary DNA (cDNA) was amplified from HeLa cDNA by PCR. After tagging with the HA sequence, the cDNA was cloned in pMXs-IRES-puro retroviral vector. The sequence coding tfLC3 cDNA was also PCR amplified using ptfLC3 vector as a template (19) and then cloned into pMXs-IRES-puro. Plat-E cells were transiently transfected with the retrovirus vector with pCG-VSV-G using PEI MAX (Polysciences; catalog no.: 24765-100). After 48 h of culturing, the culture supernatant was collected and cleared with a 0.22 μm syringe filter. HeLa cells were infected with the recombinant virus in the presence of 10 μg/ml of polybrene for 24 h. The infected cells were selected with 1 μg/ml of puromycin for three passages. The following primers were used for the construction of the LIPA gene expression vector. Forward: 5′ TTTTC TCGAG GCCAC CATGA AAATG CGGTT CTTGG GGTTG GTGG 3′, Reverse: 5′ TTTTG CGGCC GCTCA AGCGT AATCT GGAAC ATCGT ATGGG TAGCT GCCGC CGCCG CCCTG ATATT TCCTC ATTAG ATTAA 3′.

### Western blotting

Samples were separated by SDS-PAGE and transferred to polyvinylidene difluoride membranes (Millipore; catalog no.: IPVH00010). Blocking was performed with 1% (w/v) skimmed milk in Tris-buffered saline with Tween-20 (TBST) for 30 min. The membranes were then incubated with primary antibodies for 1 h. After washing with TBST, the membranes were incubated with a horseradish peroxidase–conjugated secondary antibody for 1 h. Following another round of TBST washing, the bands were visualized using a ChemiDoc Touch system (Bio-Rad) and Lumina Forte Western Horseradish Peroxidase Substrate (Millipore; catalog no.: WBLUF0100). Signal quantification was performed using ImageJ software (National Institutes of Health). The antibodies used in the study are listed in Table S2.

### Immunocytochemistry

Cells were grown on coverslips for 24 h before treatment. They were washed with PBS and then fixed in 4% paraformaldehyde (PFA) in PBS for 20 min. After fixation, the cells were permeabilized with 50 μg/ml digitonin in PBS for 10 min. The coverslips were then incubated with primary antibodies for 1 h, followed by two washes with PBS. Next, the coverslips were incubated with appropriate secondary antibodies and 4′,6-diamidino-2-phenylindole (DAPI) for 1 h. Finally, the cells were mounted on glass slides and visualized under a confocal microscope, LSM 700 confocal microscope (Zeiss). Image analysis was performed using the ImageJ software. The antibodies used in the study are listed in Table S2.

### Autophagic flux assay

Autophagic flux assays were conducted following the guidelines for autophagy assay (18, 51). Briefly, cells at 100% confluency were washed with PBS twice and treated with EBSS with or without 125 nM BafA1 (Cayman Chemicals; catalog no.: 11038) for 2 h. The cells were then washed with PBS twice and lysed in Laemmli sample buffer. The samples were analyzed by Western blotting. For nutrient depletion, the following media were used for autophagic flux assays: EBSS (Sigma–Aldrich; catalog no.: E2888), Eagle’s minimal essential medium (Sigma–Aldrich; catalog no.: M4655), DMEM (−Arg, −Leu, and −Lys) (Sigma–Aldrich; catalog no.: D9443), DMEM (low glucose, −l-Gln) (Sigma–Aldrich; catalog no.: D5921), and DMEM (−l-Gln) (Sigma–Aldrich; catalog no.: D1145). Immunoblot signals for LC3, p62, or β-actin (ACTB) were quantified using ImageJ software. After normalizing the LC3 or p62 amount to ACTB, the flux was calculated as follows: LC3 (BafA1(+) − BafA1(−))/LC3 BafA1(+).

### Measurement of autophagic flux with HaloTag fusion LC3

Halo-LC3 stable expression cells were generated by viral transduction. The pMRX-IP-HaloTag7-LC3 was used for gene transduction. Cells were incubated in DMEM with 100 nM TMR-conjugated Halo ligand (Promega; catalog no.: G8251) for 1 h. Western blotting with an anti-HaloTag antibody (Promega; catalog no.: G9211) was performed to assess the amount of Halo-LC3 and its degradation products. pMRX-IP-HaloTag7-LC3 was a gift from Noboru Mizushima (Addgene plasmid; catalog no.: 184899; http://n2t.net/addgene:184899; Research Resource Identifer: Addgene_184899) (17).

### tfLC3 assay

tfLC3 assays were performed as previously described (19). Briefly, the tfLC3 construct was virally transduced into cells to have stably tfLC3-expressing cells. After several passages, cells were selected under puromycin and cloned by limited dilution. Cells expressing tfLC3 were grown on coverslips for 24 h. The cells were then treated with EBSS with or without BafA1 for 2 h. After fixation with PFA, the cells were stained with DAPI and subsequently mounted on slide glass. The tfLC3 and DAPI signals were captured under LSM 700 and IX83 fluorescence microscope (Olympus). Image analysis was performed using the ImageJ software.

### LC3-dot flux assay

Cells were grown on coverslips for 24 h. Cells were treated or untreated with BafA1 for 2 h under DMEM or EBSS. Then, cells were washed with PBS and fixed in 4% PFA in PBS for 20 min. After fixation, the cells were permeabilized with 50 μg/ml digitonin in PBS for 10 min. The coverslips were then incubated with primary antibodies for 1 h, followed by two washes with PBS. Next, the coverslips were incubated with appropriate secondary antibodies and DAPI for 1 h. Finally, the cells were mounted on glass slides and visualized under the Olympus IX83 fluorescence microscope (Olympus). Image analysis was performed using the ImageJ software. The antibodies used in the study are listed in Table S2.

### LysoTracker staining

To monitor acidity in lysosomes, LysoTracker Red DND-99 (Invitrogen; catalog no.: L7528) was used (52). Cells grown on coverslips were treated with drugs in EBSS for 1.5 h, and then LysoTracker Red DND-99 was added to the medium at a final concentration of 200 nM. After a 30 min treatment with LysoTracker Red DND-99 in drug-containing EBSS, the cells were washed with PBS and fixed in 4% PFA in PBS for 20 min. Subsequently, the cells were permeabilized with 50 μg/ml digitonin in PBS for 10 min. The coverslips were first incubated with an anti-LAMP2 antibody for 1 h, followed by two washes with PBS. Then, the coverslips were incubated with secondary antibodies and DAPI for 1 h. The cells were mounted on glass slides and visualized under a confocal microscope, LSM 700. Image analysis was performed using the ImageJ software.

### Measurements of lysosomal pH

In accordance with the previous literature, the lysosomal pH was measured using LysoSensor Yellow/Blue dextran, 10,000 molecular weight (MW) (53, 54). Cells were cultured on glass bottom dishes and treated with 0.5 mg/ml LysoSensor Yellow/Blue dextran, 10,000 MW, and 0.1 mg/ml Dextran, Texas Red, 3000 MW for 24 h to label lysosomes. They were then washed with PBS twice and incubated in DMEM, EBSS, or EBSS containing 5 mM NH_4_Cl for 2 h. Immediately, the cells were visualized under a confocal microscope, LSM 700. As standards, cells were treated for 15 min in three different Mes buffers at pH 4.0, 5.0, and 6.0 containing nigericin and monensin. The pH in lysosomes was calculated from the ratio of blue and yellow signals in the images. Texas Red, 3000 MW was used for counterstains.

### Measurements of cellular cholesterol level

Cellular cholesterol levels were measured using the Amplex Red Cholesterol Assay Kit (Invitrogen; catalog no.: A12216) according to the manufacturer's protocol. The cells were lysed in water by sonication, and the protein amount was determined using the Bradford protein assay. For the measurements, 25 μg of protein was used.

### Lysosomal enzyme assay

Activities of lysosomal enzymes were measured using artificial 4-methylumbelliferyl substrates. Cells were extracted in water, and protein concentration was adjusted to 2, 0.2, and 0.02 mg/ml. Lysates were incubated with artificial substrates in acidic phosphate or citrate buffer at 37 °C, and reactions were immediately stopped by adding a stop solution (1 M glycine/0.2 M NaOH). For the measurement of LIPA activity, 0.345 mM 4-MU palmitate (Cayman Chemical; catalog no.: 17695-48-6) was incubated with 2 mg/ml cell extract in 100 mM sodium acetate buffer (pH 4.0) containing 1.0% Triton X-100 and 0.5% cardiolipin at 37 °C for 24 h. About 3 μM Lalistat2 was used to inhibit LIPA activity, and the difference in 4-MU between the presence and absence of Lalistat2 was calculated as LIPA activity. The fluorescence of excitation at 365 nm/emission at 450 nm was measured with a microplate reader. Each reaction condition and substrate are listed in Table S3. 4-Methylumbelliferone (Merk; catalog no.: M1381) was used for the standard.

### Measurements of lysosomal area or diameter

Measurements of lysosomal area or diameter were performed using ImageJ. Lysosomal area was calculated by analyzing particles for binarized images.

### Electron microscopy

Cells were rinsed with phosphate buffer and then fixed with 2.5% glutaraldehyde in phosphate buffer for 120 min. Subsequently, the cells were postfixed with 1% osmium tetroxide for 60 min at 4 °C. After dehydration through a graded ethanol series, the cells were embedded in Epon-resin for 3 days at 60 °C. Ultrathin sections of 70 nm were cut using an ultramicrotome (EM UC7; Leica) and mounted on mesh grids. The sections were stained with 2% uranyl acetate for 20 min at room temperature, followed by lead citrate staining for 5 min at room temperature. After drying, the samples were examined using a transmission electron microscope, JEM-1400 (JEOL), at an accelerating voltage of 80 kV.

### Cholesterol treatment

Cells were cultured in DMEM containing cholesterol lipid concentrate (Gibco; catalog no.: 12531018) at a 1× concentration (1/250 dilution) for 24 h before being used for experiments.

### Statistical analysis

Three independent experiments were performed for each dataset. The results are presented as the mean ± SD unless stated otherwise. The significance of differences was assessed using the Student's *t* test or the chi-square test, and *p* values less than 0.05 were considered significant. Multiple comparisons after one-way ANOVA were performed using Tukey's test, and *p* values less than 0.05 were considered as statistically significant. The IC_50_ curves were fitted using a four-parameter log-logistic curve with the drc package in R using the relative autophagic flux value of the untreated group as 100%.

## Data availability

All data generated or analyzed during this study are included in this published article and its supporting information file.

## Supporting information

This article contains supporting information.

## Conflict of interest

The authors declare that they have no conflicts of interest with the contents of this article.

## References

[1] Ferreira C.R., Gahl W.A. (2017). Lysosomal storage diseases. Transl. Sci. Rare Dis..

[2] Platt F.M., d'Azzo A., Davidson B.L., Neufeld E.F., Tifft C.J. (2018). Lysosomal storage diseases. Nat. Rev. Dis. Primers.

[3] Meikle P.J., Hopwood J.J., Clague A.E., Carey W.F. (1999). Prevalence of lysosomal storage disorders. JAMA.

[4] Aguisanda F., Thorne N., Zheng W. (2017). Targeting wolman disease and cholesteryl ester storage disease: disease pathogenesis and therapeutic development. Curr. Chem. Genom. Transl. Med..

[5] Hoffman E.P., Barr M.L., Giovanni M.A., Murray M.F., Adam M.P., Mirzaa G.M., Pagon R.A., Wallace S.E., Bean L.J.H., Gripp K.W. (1993). Lysosomal Acid Lipase Deficiency in GeneReviews((R)).

[6] Zhang H. (2018). Lysosomal acid lipase and lipid metabolism: new mechanisms, new questions, and new therapies. Curr. Opin. Lipidol..

[7] Pfeffer S.R. (2019). NPC intracellular cholesterol transporter 1 (NPC1)-mediated cholesterol export from lysosomes. J. Biol. Chem..

[8] Vanier M.T. (2010). Niemann-pick disease type C. Orphanet. J. Rare Dis..

[9] Sarkar S., Carroll B., Buganim Y., Maetzel D., Ng A.H., Cassady J.P. (2013). Impaired autophagy in the lipid-storage disorder Niemann-Pick type C1 disease. Cell Rep..

[10] Liedtke M., Volkner C., Hermann A., Frech M.J. (2022). Impact of organelle transport deficits on mitophagy and autophagy in niemann-pick disease Type C. Cells.

[11] Tamura A., Yui N. (2015). beta-Cyclodextrin-threaded biocleavable polyrotaxanes ameliorate impaired autophagic flux in Niemann-Pick type C disease. J. Biol. Chem..

[12] Kabeya Y., Mizushima N., Ueno T., Yamamoto A., Kirisako T., Noda T. (2000). LC3, a mammalian homologue of yeast Apg8p, is localized in autophagosome membranes after processing. EMBO J..

[13] Bowman E.J., Siebers A., Altendorf K. (1988). Bafilomycins: a class of inhibitors of membrane ATPases from microorganisms, animal cells, and plant cells. Proc. Natl. Acad. Sci. U. S. A..

[14] Werner G., Hagenmaier H., Drautz H., Baumgartner A., Zahner H. (1984). Metabolic products of microorganisms. Bafilomycins, a new group of macrolide antibiotics. Production, isolation, chemical structure and biological activity. J. Antibiot. (Tokyo).

[15] Wang R., Wang J., Hassan A., Lee C.H., Xie X.S., Li X. (2021). Molecular basis of V-ATPase inhibition by bafilomycin A1. Nat. Commun..

[16] Alers S., Loffler A.S., Wesselborg S., Stork B. (2012). Role of AMPK-mTOR-Ulk1/2 in the regulation of autophagy: cross talk, shortcuts, and feedbacks. Mol. Cell Biol..

[17] Yim W.W., Yamamoto H., Mizushima N. (2022). A pulse-chasable reporter processing assay for mammalian autophagic flux with HaloTag. Elife.

[18] Yu L., Chen Y., Tooze S.A. (2018). Autophagy pathway: cellular and molecular mechanisms. Autophagy.

[19] Kimura S., Noda T., Yoshimori T. (2007). Dissection of the autophagosome maturation process by a novel reporter protein, tandem fluorescent-tagged LC3. Autophagy.

[20] Imamoto Y., Tanaka H., Takahashi K., Konno Y., Suzawa T. (2013). Advantages of AlaGln as an additive to cell culture medium: use with anti-CD20 chimeric antibody-producing POTELLIGENT CHO cell lines. Cytotechnology.

[21] Arii K., Kai T., Kokuba Y. (1999). Degradation kinetics of L-alanyl-L-glutamine and its derivatives in aqueous solution. Eur. J. Pharm. Sci..

[22] Montevecchi G., Masino F., Antonelli A. (2010). Pyroglutamic acid development during grape must cooking. Eur. Food Res. Technol..

[23] Dikic I., Elazar Z. (2018). Mechanism and medical implications of mammalian autophagy. Nat. Rev. Mol. Cell Biol..

[24] Soria L.R., Brunetti-Pierri N. (2019). Ammonia and autophagy: an emerging relationship with implications for disorders with hyperammonemia. J. Inherit. Metab. Dis..

[25] Fedele A.O., Proud C.G. (2020). Chloroquine and bafilomycin A mimic lysosomal storage disorders and impair mTORC1 signalling. Biosci. Rep..

[26] Colacurcio D.J., Nixon R.A. (2016). Disorders of lysosomal acidification-The emerging role of v-ATPase in aging and neurodegenerative disease. Ageing Res. Rev..

[27] Abuammar H., Bhattacharjee A., Simon-Vecsei Z., Blastyak A., Csordas G., Pali T. (2021). Ion channels and pumps in autophagy: a reciprocal relationship. Cells.

[28] Pamarthy S., Kulshrestha A., Katara G.K., Beaman K.D. (2018). The curious case of vacuolar ATPase: regulation of signaling pathways. Mol. Cancer.

[29] Li Z., Ji X., Wang W., Liu J., Liang X., Wu H. (2016). Ammonia induces autophagy through dopamine receptor D3 and MTOR. PLoS One.

[30] Lemieux B., Percival M.D., Falgueyret J.P. (2004). Quantitation of the lysosomotropic character of cationic amphiphilic drugs using the fluorescent basic amine Red DND-99. Anal. Biochem..

[31] Mindell J.A. (2012). Lysosomal acidification mechanisms. Annu. Rev. Physiol..

[32] Cabrera-Reyes F., Parra-Ruiz C., Yuseff M.I., Zanlungo S. (2021). Alterations in lysosome homeostasis in lipid-related disorders: impact on metabolic tissues and immune cells. Front. Cell Dev. Biol..

[33] Settembre C., Fraldi A., Jahreiss L., Spampanato C., Venturi C., Medina D. (2008). A block of autophagy in lysosomal storage disorders. Hum. Mol. Genet..

[34] Rocha N., Kuijl C., van der Kant R., Janssen L., Houben D., Janssen H. (2009). Cholesterol sensor ORP1L contacts the ER protein VAP to control Rab7-RILP-p150 Glued and late endosome positioning. J. Cell Biol..

[35] Velho R.V., Harms F.L., Danyukova T., Ludwig N.F., Friez M.J., Cathey S.S. (2019). The lysosomal storage disorders mucolipidosis type II, type III alpha/beta, and type III gamma: update on GNPTAB and GNPTG mutations. Hum. Mutat..

[36] Otomo T., Higaki K., Nanba E., Ozono K., Sakai N. (2011). Lysosomal storage causes cellular dysfunction in mucolipidosis II skin fibroblasts. J. Biol. Chem..

[37] Patterson M., Adam M.P., Feldman J., Mirzaa G.M., Pagon R.A., Wallace S.E., Bean L.J.H. (1993). Niemann-Pick Disease Type C in GeneReviews((R)).

[38] Poole B., Ohkuma S. (1981). Effect of weak bases on the intralysosomal pH in mouse peritoneal macrophages. J. Cell Biol..

[39] Xiong J., Zhu M.X. (2016). Regulation of lysosomal ion homeostasis by channels and transporters. Sci. China Life Sci..

[40] Weiner I.D., Mitch W.E., Sands J.M. (2015). Urea and ammonia metabolism and the control of renal nitrogen excretion. Clin. J. Am. Soc. Nephrol..

[41] Dimski D.S. (1994). Ammonia metabolism and the urea cycle: function and clinical implications. J. Vet. Intern. Med..

[42] Sakaguchi C. (1965). Metabolism of ammonia under various environmental conditions 1. Chages in the ammonia content of brain, liver, and blood under some environmental conditions. Nihon Eiseigaku Zasshi.

[43] Auwerda J.J., Leebeek F.W., Wilson J.H., van Diggelen O.P., Lam K.H., Sonneveld P. (2006). Acquired lysosomal storage caused by frequent plasmapheresis procedures with hydroxyethyl starch. Transfusion.

[44] Shayman J.A., Abe A. (2013). Drug induced phospholipidosis: an acquired lysosomal storage disorder. Biochim. Biophys. Acta.

[45] Yamamoto T., Takabatake Y., Takahashi A., Kimura T., Namba T., Matsuda J. (2017). High-fat diet-induced lysosomal dysfunction and impaired autophagic flux contribute to lipotoxicity in the kidney. J. Am. Soc. Nephrol..

[46] Rampanelli E., Ochodnicky P., Vissers J.P., Butter L.M., Claessen N., Calcagni A. (2018). Excessive dietary lipid intake provokes an acquired form of lysosomal lipid storage disease in the kidney. J. Pathol..

[47] Khan K., Elia M. (1991). Factors affecting the stability of L-glutamine in solution. Clin. Nutr..

[48] Jagusic M., Forcic D., Brgles M., Kutle L., Santak M., Jergovic M. (2016). Stability of minimum essential medium functionality despite L-glutamine decomposition. Cytotechnology.

[49] Saftig P., Klumperman J. (2009). Lysosome biogenesis and lysosomal membrane proteins: trafficking meets function. Nat. Rev. Mol. Cell Biol..

[50] Pu J., Guardia C.M., Keren-Kaplan T., Bonifacino J.S. (2016). Mechanisms and functions of lysosome positioning. J. Cell Sci..

[51] Klionsky D.J., Abdel-Aziz A.K., Abdelfatah S., Abdellatif M., Abdoli A., Abel S. (2021). Guidelines for the use and interpretation of assays for monitoring autophagy (4th edition). Autophagy.

[52] Holland P., Torgersen M.L., Sandvig K., Simonsen A. (2014). LYST affects lysosome size and quantity, but not trafficking or degradation through autophagy or endocytosis. Traffic.

[53] Ma L., Ouyang Q., Werthmann G.C., Thompson H.M., Morrow E.M. (2017). Live-cell microscopy and fluorescence-based measurement of luminal pH in intracellular organelles. Front. Cell Dev. Biol..

[54] DePedro H.M., Urayama P. (2009). Using LysoSensor Yellow/Blue DND-160 to sense acidic pH under high hydrostatic pressures. Anal. Biochem..

