# Supplementary material for: Impaired lysosomal acidity maintenance in acid lipase-deficient cells leads to defective autophagy
Source: J Biol Chem. 2024 Feb 12;300(3):105743. doi: 10.1016/j.jbc.2024.105743 (PMC10933554; doi:10.1016/j.jbc.2024.105743)
Supplement: Supporting Figures S1–S6 and Tables S1–S3 [file mmc1.pdf]

Supporting Information

Impaired lysosomal acidity maintenance in acid lipase-deficient cells leads to defective autophagy

Takahito Moriwaki, Seigo Terawaki, Takanobu Otomo

Table of contents

- Figure S1
- Figure S2
- Figure S3
- Figure S4
- Figure S5
- Figure S6
- Table S1
- Table S2
- Table S3

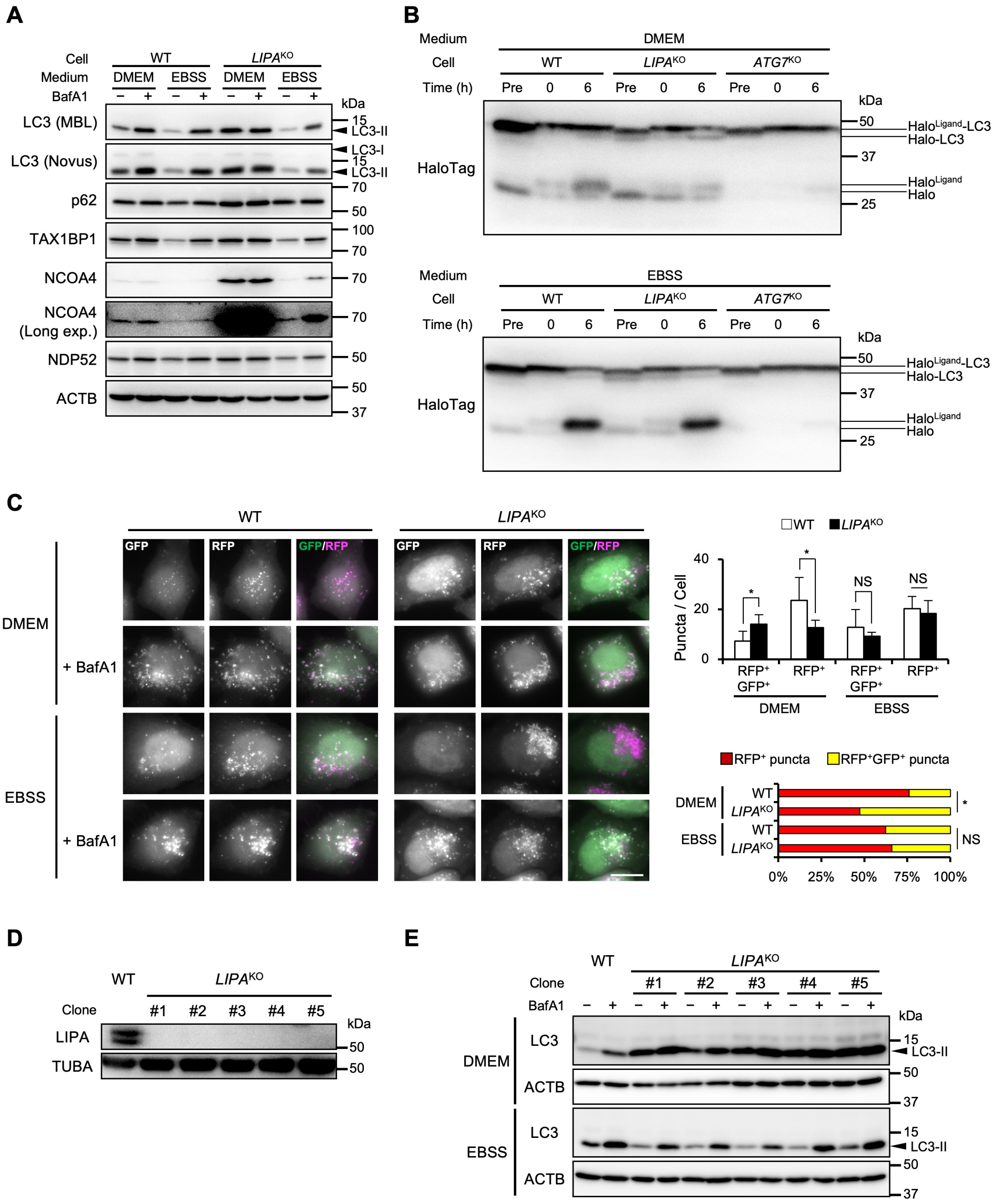

Figure S1. Impaired autophagy in *LIPA*^{KO} cell lines.

A, Autophagic flux of various substrates. Cells were treated with BafA1 under nutrient-rich (DMEM) or starved (EBSS) conditions for 2 h and autophagic flux was assessed by western blotting with the indicated antibody. We used the anti-LC3 antibody from the MBL company throughout the manuscript, indicated as "LC3".

Here we show the comparison of different antibodies against LC3. *B*, LC3 flux assay using HaloTag-fused LC3. Cells stably expressing Halo-LC3 were treated with 100 nM TMR-conjugated Halo ligand for 1 h, and then chased in DMEM or EBSS for 6 h. Pre-treatment of ligand-bound HaloTag ($Halo^{Ligand}$) (Pre), post-treatment of $Halo^{Ligand}$ (0), and after 6 h culture (6) was analyzed by western blotting using the anti-HaloTag antibody. *ATG7*^{KO} cells were used as a control of autophagy deficiency. *C*, tfLC3 assay. The numbers of RFP^+ puncta and RFP^+GFP^+ puncta were counted in both DMEM and EBSS. Representative images (left), the average number of puncta (upper right), and the ratio of them (lower right) were shown. Mean and SD counts were obtained from 5 pictures and analyzed statistically by Student's *t*-test (* $p < 0.05$, NS $p \geq 0.05$). The average ratios of puncta were statistically analyzed by the chi-square test (* $p < 0.05$, NS $p \geq 0.05$). Scale bar = 10 µm. *D,* Depletion of LIPA in the *LIPA*^{KO} cells used in the present study. LIPA protein was confirmed by western blotting. All knockout clones exhibited the absence of LIPA protein. *E*, Assessment of autophagic flux in multiple knockout *LIPA*^{KO} cell lines under both DMEM and EBSS. All knockout clones demonstrated impaired autophagy under nutrient-rich conditions and restored autophagy under starved conditions.

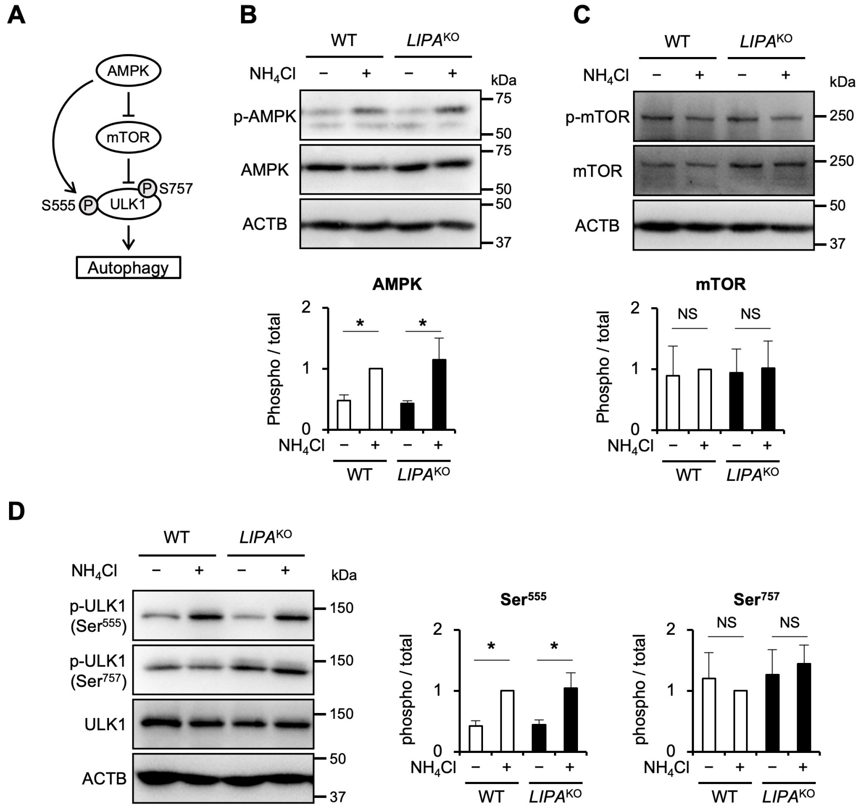

Figure S2. Normal Induction of autophagy in *LIPA*^{KO} cells.

A, Schematic representation of upstream signaling in autophagy. ULK1 is a key kinase involved in autophagy initiation. Its activity is directly regulated by phosphorylation at Ser^{757} by mTOR (negative regulation) and at Ser^{555} by AMPK (positive regulation), or indirectly by AMPK through modulation of mTOR function (positive regulation). *B-D*, Phosphorylation status of key signaling molecules upstream of autophagy in wild-type and *LIPA*^{KO} cells. The phosphorylation levels of key signaling molecules upstream of autophagy were determined in wild-type and *LIPA*^{KO} cells cultured in EBSS containing 0 or 5 mM NH_4Cl for 2 h using western blotting and evaluated by densitometry for *B* (AMPK), *C* (mTOR), and *D* (ULK1). The data represents the mean and SD from three independent experiments. Statistical analyses were performed using multiple comparison one-way ANOVA followed by post-hoc Tukey test (*$p < 0.05$).

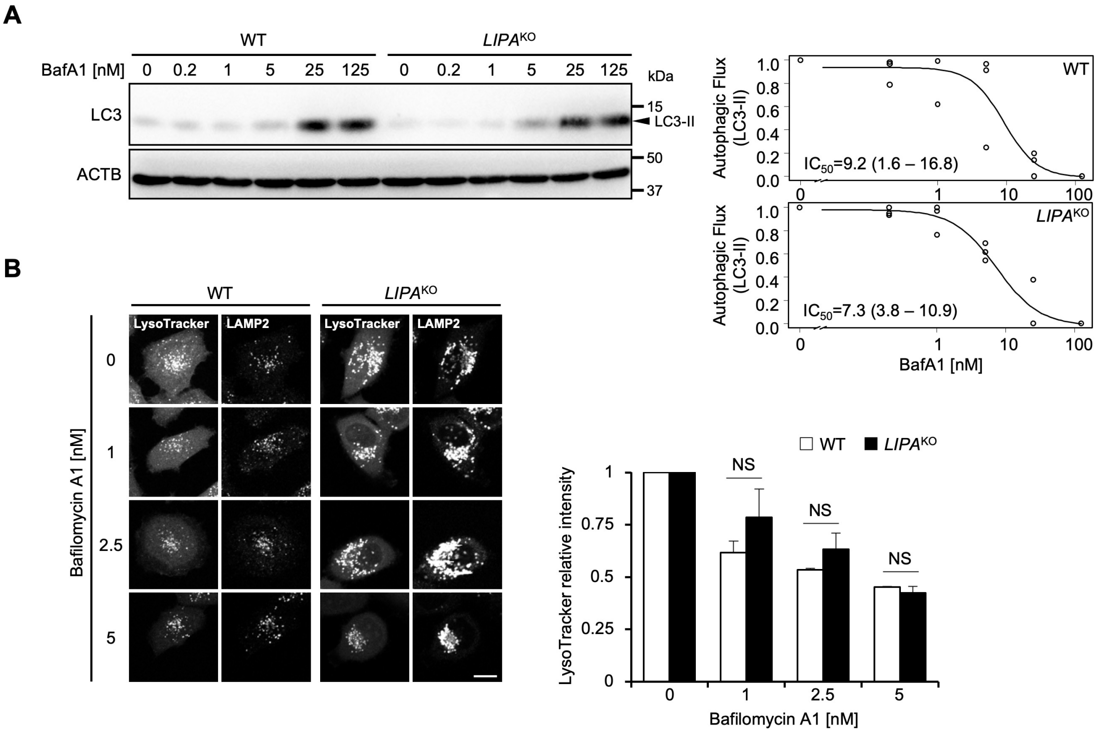

Figure S3. Comparable sensitivity to BafA1 in *LIPA*^{KO} cells as WT.

A, Determination of IC_{50} for BafA1. Cells were treated with BafA1 at the indicated doses in EBSS for 2 h, and LC3-II flux was assessed through western blotting. Representative images (left) and a four-parameter log-logistic curve from three independent experiments (right) are provided. Open circles represent relative LC3-II flux at each concentration of BafA1 in three independent experiments. *B*, Effects of BafA1 on lysosomal pH. Cells were treated with the indicated concentrations of BafA1 for 2 h in EBSS. Lysosomes were stained with LysoTracker™ Red for 30 min, followed by LAMP2 immunostaining. At least 10 pictures were analyzed in each condition. The graph represents the mean and SD from three independent experiments. Scale bar = 10 µm.

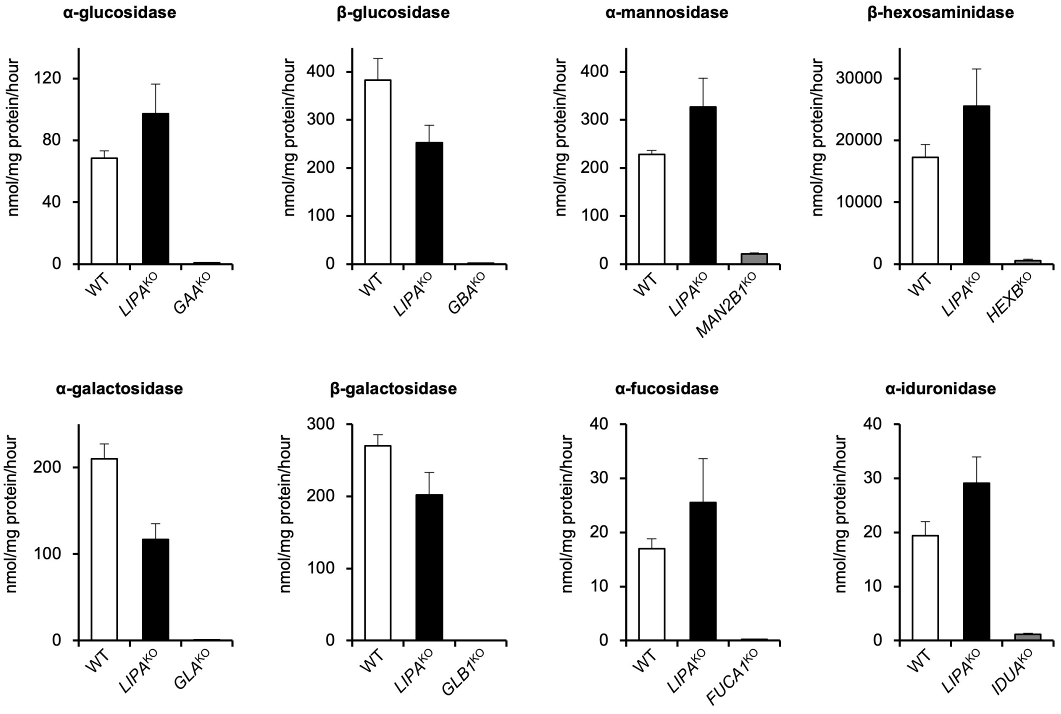

Figure S4. Maintenance of normal levels of lysosomal enzymes in *LIPA*^{KO} cells.

Lysosomal enzyme levels were assessed using 4-MU-conjugated substrates, with knockout cells lacking each respective enzyme gene serving as negative controls. The graph represents the mean and SD from three independent experiments.

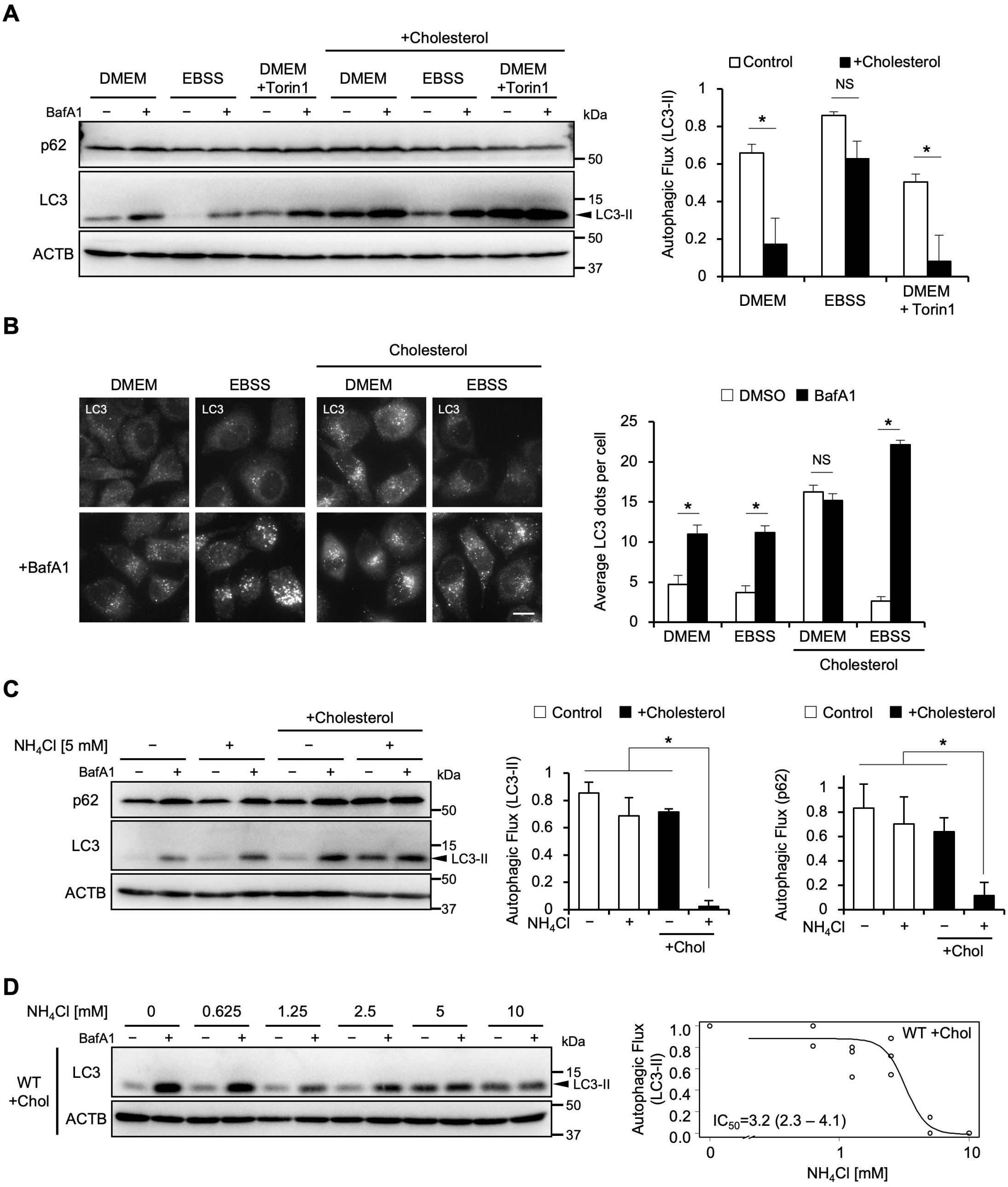

Figure S5. Reproduction of phenotype of *LIPA*^KO^ cells by cholesterol treatment. *A,* Determination of autophagic flux in cholesterol-treated cells. Wild-type cells were pre-treated with cholesterol for 24 h, and then the LC3-II and p62 autophagic flux was measured under nutrient-rich, starve, and nutrient-rich +Torin1. A representative immunoblot is displayed on the left side, while quantitative analyses of relative flux are presented on the right side. *B*, LC3-dot flux assay. Cells were

stained with anti-LC3 and visualized by fluorescence microscopy. Typical images (left) and quantified results (right) were shown. The average LC3 dot count per cell was obtained from 10 pictures in three independent experiments. *C*, Determination of autophagic flux under EBSS containing 5 mM NH_4Cl. Wild-type cells were pre-treated with cholesterol for 24 h, and then the LC3-II and p62 autophagic flux was measured under EBSS containing 5 mM NH_4Cl. A representative immunoblot is displayed on the left side, while quantitative analyses of relative flux are presented on the right side. The graph represents the mean and SD from three independent experiments. *D*, IC_{50} for NH_4Cl in cholesterol-treated cells. Wild-type cells were cultured in the presence of cholesterol for 24 h. The cells were subsequently treated with the indicated concentration of NH_4Cl for 2 h in EBSS, and the susceptibility of autophagy to NH_4Cl was evaluated using the LC3-II flux assay. Representative blots are shown, and the dose-response curve and IC_{50} for NH_4Cl were calculated from three independent experiments. Open circles represent relative LC3-II flux at each concentration of NH_4Cl, and IC_{50} is reported as IC_{50} (95% CI).

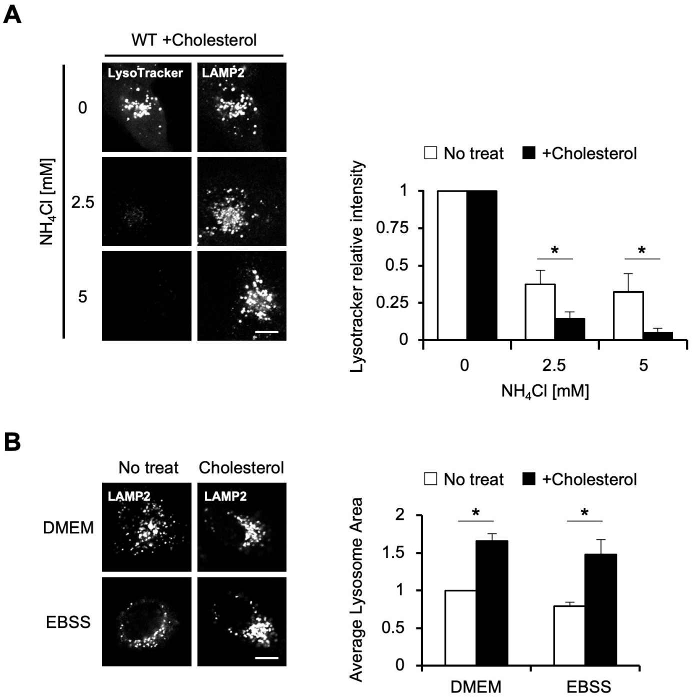

Figure S6. Decreased lysosomal acidity maintenance and increased lysosomal size in cholesterol-treated WT cells.

A, Determination of decreased pH treated with NH_4Cl. Cells were treated with the indicated concentration of NH_4Cl for 2 h in EBSS. Lysosomes were stained with LysoTracker™ Red for 30 min, followed by LAMP2 immunostaining. The relative LysoTracker™ intensities on lysosomes (LAMP2 positive) were quantified using ImageJ. Image analysis was conducted on a minimum of 10 pictures in each condition. *B*, Determination of lysosomal size. Wild-type cells, either non-treated or treated with cholesterol for 24 h, were cultured in DMEM or EBSS for 2 h. Then, lysosomes were visualized by immunostaining of LAMP2. The average size of LAMP2-positive vesicles was measured using ImageJ. A total of 10 pictures were analyzed in each condition. All data represent the mean and SD from three independent experiments. Statistical analyses were performed using multiple comparison one-way ANOVA followed by post-hoc Tukey test (*$p < 0.05$).

Gene name (Related disease)		Mutations		Sequence of crRNA+PAM in guide RNA (5' to 3')
		allele 1	allele 2	
FUCA1 (α-fucosidosis)		del 1 bp	homo	GAACTTGGCTTCGTCGAACCAGG
GAA (Pompe disease)		del 2 bp	homo	CCCGCGGCTGTTGCTACATCCCT
GBA1 (Gaucher disease)		del 4 bp	del 5 bp	AGACCAATGGAGCGGTGAATGGG
GLA (Fabry disease)		del 1 bp	homo	GCTAGCTGGCGAATCCCATGAGG
GLB1 (Morquio syndrome B)		ins 2 bp	del 19 bp	CCAGTAGAAGCGGGGCACACGGG
GNPTAB (MLII/I-cell disease)		del 2 bp	del 8 bp	CCACAGGTATGGGCTCTACGTGT
HEXB (Sandhoff's disease)		del 1 bp	homo	CCCAATTCCACGGCGGGCCCCTC
IDUA (Hurler syndrome)		ins 1 bp	homo	CCCTGCGGCGCTTCTGGAGGAGC
LIPA (Wolman's disease, cholesteryl ester storage disease)	#1<br>#2<br>#3<br>#4<br>#5	del 1 bp<br>del 4 bp<br>del 13 bp<br>ins 1 bp<br>ins 1 bp	homo<br>homo<br>homo<br>del 5 bp<br>del 5 bp	TCCCATGAGGAATTCGGTTAAGG
MAN2B1 (α-mannosidosis)		del 2 bp	homo	ACGTAAATGAAGCGACGGGTGGG

Table S1. Cell lines used in this study.

The knockout cell lines were generated using the CRISPR/Cas9. Target genes and their associated diseases are presented along with the mutations introduced through gene targeting. Additionally, the sequences of crRNA and the corresponding PAM sequences used for each knockout cell line are provided, with the PAM sequence indicated by underlining. Each mutation was confirmed through Sanger sequencing. For the majority of the experiments in the present study, clone #1 of *LIPA*^{KO} was primarily employed unless otherwise specified.

Target protein	Company	Catalog number	Species
ACTB	MBL	M177-3	Mouse
AMPKα	BioLegend	600551	Mouse
AMPKα Phospho (Thr^{172})	BioLegend	600651	Mouse
ATP6V0D1	Proteintech	18274-1-AP	Rabbit
ATP6V1B1/2	Santa Cruz	sc-55544	Mouse
Halo-tag	Promega	G9211	Mouse
LIPA	Santa Cruz	sc-58374	Mouse
LAMP1	BioLegend	328602	Mouse
LAMP2	BioLegend	354302	Mouse
LC3	MBL	PM036	Rabbit
LC3	Novus	NB600-1384	Rabbit
mTOR	BioLegend	659201	Mouse
mTOR Phospho (Ser^{2448})	BioLegend	610301	Mouse
NCOA4	SIGMA-ALDRICH	SAB1404569	Mouse
NDP52	GeneTex	GTX115378	Rabbit
p62	MBL	PM045	Rabbit
TAX1BP1	Proteintech	14424-1-AP	Rabbit
TUBA	Santa Cruz	sc-32293	Mouse
ULK1	Cell Signaling Technology	8054	Rabbit
ULK1 Phospho (Ser^{555})	Cell Signaling Technology	5869	Rabbit
ULK1 Phospho (Ser^{757})	Cell Signaling Technology	6888	Rabbit

Target protein	modification	Company	Catalog number	Species
Rabbit IgG	Alexa Fluor™ 488	Thermo Fisher Scientific	A21206	Donkey
Mouse IgG	Alexa Fluor™ 647	Thermo Fisher Scientific	A31571	Donkey
Mouse IgG (H+L)	HRP	Thermo Fisher Scientific	31430	Goat
Rabbit IgG (H+L)	HRP	Thermo Fisher Scientific	31460	Goat

Table S2. Antibodies used in the current study.

The antibodies used for western blotting or immunocytochemistry are shown.

Enzyme	Reaction Mix	Reaction
α-fucosidase	0.5 mM 4-MU α-L-fucopyranoside<br>(Merk; catalog no.: M8527)<br>C-P buffer (pH=5.2)	37 °C, 1 h
α-galactosidase	2.5 mM 4-MU α-D-galactopyranoside<br>(Merk; catalog no.: M7633)<br>20 mM N-Acetyl-D-galactosamine<br>C-P buffer (pH=4.4)	37 °C, 1 h
α-glucosidase	1 mM 4-MU α-D-glucopyranoside<br>(Merk; catalog no.: 69591)<br>C-P buffer (pH=4.0)	37 °C, 1 h
α-iduronidase	0.8 mM 4-MU α-L-iduronide<br>(Glycosynth; catalog no.: 44076)<br>30 mM NaCl<br>80 mM Formate buffer (pH 3.5)	37 °C, 1 h
α-mannosidase	2.5 mM 4-MU α-D-mannopyranoside<br>(Merk; catalog no.: M3657)<br>C-P buffer (pH=4.4)	37 °C, 1 h
β-glucosidase	2.5 mM 4-MU β-D-glucopyranoside<br>(Merk; catalog no.: M3633)<br>0.25 % Sodium Taurocholate<br>0.2 % TritonX-100<br>C-P buffer (pH=5.4)	37 °C, 1 h
β-hexosaminidase	1.5 mM 4-MU N-acetyl-β-D-glucosaminide<br>(Merk; catalog no.: M2133)<br>C-P buffer (pH=4.4)	37 °C, 1 h
Lysosomal acid lipase A	0.345 mM 4-MU Palmitate<br>(Cayman Chemical; catalog no.: 17695-48-6)<br>1.0 % TritonX-100<br>0.5 % cardiolipin.<br>0 or 3 μM Lalistat2<br>100 mM sodium acetate buffer (pH4.0)	37 °C, 24 h

Table S3. Experimental conditions for enzyme assays.

The reaction conditions for enzyme assays are provided. "C-P buffer" refers to Citrate-Phosphate Buffer adjusted to the indicated pH values.
